# The human resource information system: a rapid appraisal of Pakistan’s capacity to employ the tool

**DOI:** 10.1186/1472-6947-13-104

**Published:** 2013-09-10

**Authors:** Ramesh Kumar, Babar Tasneem Shaikh, Jamil Ahmed, Zulfiqar Khan, Sayed Mursalin, Mahmood Iqbal Memon, Shagufta Zareen

**Affiliations:** 1 Department of Health Systems & Policy, Health Services Academy, Islamabad, Pakistan; 2 World Health Organization, Islamabad, Pakistan; 3 Health Department, Government of Sindh, Karachi, Pakistan; 4 Public health Consultant, Lahore, Pakistan

**Keywords:** Human resources for health, Human resource information system, Health system, Pakistan

## Abstract

**Background:**

Human resources are an important building block of the health system. During the last decade, enormous investment has gone into the information systems to manage human resources, but due to the lack of a clear vision, policy, and strategy, the results of these efforts have not been very visible. No reliable information portal captures the actual state of human resources in Pakistan’s health sector. The World Health Organization (WHO) has provided technical support for the assessment of the existing system and development of a comprehensive Human Resource Information System (HRIS) in Pakistan.

**Methods:**

The questions in the WHO-HRIS Assessment tool were distributed into five thematic groups. Purposively selected (n=65) representatives from the government, private sector, and development partners participated in this cross sectional study, based on their programmatic affiliations.

**Results:**

Fifty-five percent of organizations and departments have an independent Human Resources (HR) section managed by an establishment branch and are fully equipped with functional computers. Forty-five organizations (70%) had HR rules, regulations and coordination mechanisms, yet these are not implemented. Data reporting is mainly in paper form, on prescribed forms (51%), registers (3%) or even plain papers (20%). Data analysis does not give inputs to the decision making process and dissemination of information is quite erratic. Most of the organizations had no feedback mechanism for cross checking the HR data, rendering it unreliable.

**Conclusion:**

Pakistan is lacking appropriate HRIS management. The current HRIS indeed has a multitude of problems. In the wake of 2011 reforms within the health sector, provinces are even in a greater need for planning their respective health department services and must work on the deficiencies and inefficiencies of their HRIS so that the gaps and HR needs are better aligned for reaching the 2015 UN Millennium Development Goals (MDGs) targets.

## Background

Human resources are the backbone of any national health care system. To ensure that the rightpersonnel are in the right place with the right skills, we need up to date and accurate data on human resource for health (HRH). A strong human resource information system (HRIS) helps policy makers, administrators, and managers quickly answer key questions affecting health care service delivery. It has been argued that in developing countries there are deficiencies in the availability of HRH-specific information, and even more so in with regard to its consolidation, standardization, analysis, and use in the planning and managerial processes [1]. Ideally, HRIS development would begin with a systematic review and appraisal of existing data sources, mechanisms, indicators, tools, and systems in both public and private sectors. Once HRIS assessment has been carried out, the plan would proceed in a systematic manner to make decisions concerning HR issues [2].

### Global HRIS practices

Many developing countries face daunting obstacles in meeting the health care needs of their people. One important reason is the lack of reliable and accurate data on HRH which impedes proper planning, decision making and even the resource allocation [1]. A strong HRIS informs and enables the decision makers to address many of the key policy and management questions, which of course affect the health care delivery system. The HRIS is an important tool which is globally used by the decision makers to use data for leading and managing their HR in the health sector; however, Pakistan has been an exception [2]. The World Health Organization (WHO), through its Department of Human Resources for Health, works with the member states to strengthen their capacity for managing their health workforce, so that health services can become more and more responsive. WHO and other development agencies greatly emphasize on HR development within each country’s health sector policies. Ideally, HRIS involves forging a global consensus on HRH, by means of pursuing in-depth work in countries and building networks. There are inclusive tools to analyze and address various HRH issues in order to better assist countries. These tools lay out a general framework built in collaboration with partners, including the staff of ministries of health, health training institutions, professional associations and bilateral and international partners. Information plays a vital role in an effective management of any health system. The HRIS provides specific information support to the decision makers at various levels of the health system and assist in evidence-based decision making for an effective management of the human resources for health [3]. Appropriate use of information technology in health care depends a lot on the interest and support of the leadership in health care system. The organization needs strong leadership with sufficient means and abilities to manage the change in the organizational output [4].

### HRIS and the pakistani health system

Being a low-resource country, Pakistan’s health sector is facing tremendous problems in meeting the health care needs of its people, mainly because of dearth of trained HR in the rural areas where 65% of the population lives [5]. Pakistan is considered among those 57 countries which face an acute shortage of HRH. Published data suggests that one of the contributing factors to the dearth of workforce is the absence of a functional HRIS, and the subsequent incapacity for a sound HR decision making [6]. There is a lack of a clear vision, policy, and strategy for HRH, and since the Government of Pakistan has devolved the Federal Ministry of Health after a constitutional amendment in 2011, the importance and need of a HRIS strategic plan with a clear breakdown of short term, medium term, and long term targets has become more noticeable. HRIS was never integrated within the National Health Management Information System (NHMIS), a system which was designed to generate the information on the status of ongoing health-related activities. NHMIS was supposed to facilitate evidence-based decision-making and an effective management of health care system at all levels. Traditionally, the focus in information systems has been merely on the technical aspects of the health services. However, recently the data regarding HR has been recognized as an important factor in the information systems [7].

For all the national programmes of health, implementation largely lies with the provincial governments, with an extensive network of nearly 10,000 service outlets at the district level: primary, secondary and tertiary. The Executive District Officer Health (EDO-H) is in charge of the district and is responsible for delivering promotive, preventive and curative services through the outreach workers and the primary care district facilities. The managers of all public sector hospitals in the district, report to EDO-H [8]. The efficiency of the entire system, however, ultimately depends on a robust information system, quality of data generated and the effective use of evidence for decision making [9].

The total number of health workers in public sector alone is more than 250,000. There are 448 medical schools, which produce more than 16,000 different categories of health workers [5]. These numbers, however, have been challenged multiple times and their credibility is doubtful. The HRIS in Pakistan suffers from lack of efficiency as well as usefulness. This information system also lacks coherence and integration in terms of Pakistan’s HR data gathering process [10]. After the 2011 reforms, the responsibility of data management was entirely delegated to the provinces. The HR data is now collected in the routine District Health Information System (DHIS). Therefore, the provincial governments now ought to strategize the mapping, recruitment, deployment and fair distribution of HRH, which is vital to strengthen the health system [11].

### Study objectives

With this background, the present study was designed. The objectives of the study were threefold: (i) to document as to how HR information is currently being collected, managed and reported; (ii) to identify the gaps related to HRH information that need to be urgently addressed; and (iii) to suggest the tools and processes for managing HR data.

## Methods

### Study design

This was a cross sectional quantitative study for which data was collected from the key stakeholders and informants, using a self-administered structured questionnaire. Observations and examination of HRIS records were also done simultaneously in order to extract the information required.

### Sampling frame and size

Data was collected from all the public and private institutions across the country (65 in all), managing a HRIS. A total of 65 stakeholders involved in HRIS from public and private sector; including from Karachi, Hyderabad, Tando Muhammad Khan and Jamshoro in Sindh, Quetta and Mastung in Baluchistan, Lahore, Jhang, Sheikhupura, Gujranwala, Faisalabad, Kasur and Nankana in Punjab, Mardan and Peshawar in Khyber Pukhtunkhwa /FATA province, Bagh and Muzzafarabad in Azad Jammu & Kashmir, and Islamabad. Majority of these (41) were from public sector that represented the district/ provincial health offices, tertiary level care hospitals, DHIS cell and primary health care centers; while 24 institutions were from the private sector that included hospitals, international NGOs and donors.

Principal Investigator trained the data collectors before the start the data collection process; all of them were experienced public health researchers. These data collectors then personally handed over the self-administered questionnaire to the respondents and clarified the respondents’ queries. Out of the 65 institutions surveyed, twenty-three (36%) were in Punjab Province, ten (15%) in Sindh, ten (15%) in Baluchistan, eight (12%) in Azad Jammu & Kashmir, eight (12%) Khyber Pukhtunkhwa and six (10%) represented Federal capital Islamabad. The cadres of the respondents varied from Director General of Health to Director Provincial Health Development Centers. There were Provincial Managers of the national programmes and Chiefs of HMIS cells. The survey responses also included those of local hospital administrators and senior officials representing nongovernmental organizations (NGOs) and other development organizations. However, the answers did not have much of the difference, and therefore no significant association was found with any special cadre.

### Data collection

The period of data collection was two weeks (1–15 November, 2011). The principal investigator was personally responsible for the distribution and collection of all the filled questionnaires to control and ensure the data quality. The Management Sciences for Health HRIS assessment tool was used for the data collection [12]. A self-administered questionnaire was pre-tested, and after minor adjustments mainly related to sequencing of the questions, final version was administered. The data was collected on organization information, infrastructure, HRIS software, data collection, data reporting and on the use and sustainability of HRIS. All data collected was subsequently entered in Statistical Package for Social Science (SPSS) version 17, for the purpose of analysis.

#### *Ethical considerations*

A written informed consent was obtained from all of the study participants. Ethical approval was sought from the WHO and the respective Departments of Health in all provinces of Pakistan. Information was kept confidential and anonymous during the analysis by using codes for each respondent.

## Results

A descriptive analysis of the data was conducted in order to ascertain the variety and gravity of HRIS issues. A total of 65 facilities were covered. Twenty- four district health offices, 13 large hospitals, 12 private hospitals, nine DHIS “cells” were included and the rest were primary health care centers and the donors’ organizations.

In 54 (83%) of cases, a clerk oversaw the HRIS. Twenty-five (38%) institutions reported having one computer. Twenty-six (40%) institutions reported having less than four functioning computers and fifty-five (85%) institutions reported having a functional printer. In 23 (35%) institutions, the staff members trained in HRIS numbered less than two staff members were found to be trained.

### Institutional arrangements for HRIS

When inquired about the institutions’ arrangement for running and maintaining the HRIS, it was reported that there was an independent HR section in about half of the institutions; however, most of these did not have the internet facility, and even a mobile phone device. Moreover, most organizations do not have the HRIS built in their routine Management Information System (MIS). Table 1 shows the details of institutional arrangements.

**Table 1:** Institutional arrangements for HRIS (n=65)

Independent HR Section in office	55%
Internet facility	29%
Mobile phone	18%
Separate computer room in office	54%
Backup power supply to run computers	54%
Trained personnel for HRIS	72%
HRIS as part of general MIS	20%
HRIS policy manual available	20%
HR indicators used for data collection purpose	14%
HR data bank available (software based)	22%
Type of HRIS	
• Paper based	42%
• Computerized	32%
• Mixed	26%
Frequency of updating	
• Monthly	7%
• Quarterly	89%
• Uncertain	4%

### HR Policy, planning, coordination and capacity

Only in three facilities (5%), the respondents reported to have a separate staff for HR planning. Although 45 facilities (70%) shared that HR procedures are practiced, yet a concise policy document or manual was not available (Table 2). The HR data management plan and a separate budget allocation were almost non-existent in most of the institutions (92%).

**Table 2:** HR Policy, planning, coordination and capacity (n=65)

HR rules and regulations	70%
HR policy manual	25%
Separate HR section staff at provincial level	5%
Functional HR section	20%
HR staff trained in data management	10%
HR data management training plan	8%
Separate budget for HR data management activity	0%

### Data collection system

Most of the institutions do not have an electronic HRIS, and therefore HR departments relied on the outdated methods of data collection such as prescribed forms and registers for data record. Data was updated when required by the higher ups or policy makers. In the majority of cases (94%), HR data maintenance was not the responsibility of the HR section (Table 3). Data sources were also unreliable, to a large extent. Data accuracy and quality was also not up to the mark in most of the cases. Audit of data quality is not carried out on regular basis.

**Table 3:** HR Data collection system (n=65)

HR data collection methods	Prescribed forms	51%
	Registers	3%
	Plain Papers	20%
	Others	26%
HR data maintenance	Individual files	71%
	Spread sheets	17%
	Specific software	12%
HR data maintenance frequency	Regularly	17%
	Irregularly	12%
	When required	71%
HR data maintenance responsibility	HR section	6%
	Individual officials	2%
	Separate HR staff	3%
	Admin staff	57%
	Establishment staff	32%
HR data sources	DHIS	38%
	HRIS manual	18%
	Others	32%
Indicate type of HR data collected regularly	Employee data	35%
	Payroll	45%
	Training	14%
	Other	6%
Data accuracy	Inaccurate	2%
	Somewhat accurate	38%
	Very accurate	40%
	Not sure	20%
Frequency of data quality audit	Monthly	3%
	Annually	6%
	When required	69%
	Don’t know	22%
Capacity of implementation of modern data collection system	Yes	43%
	No	51%
	Not sure	6%

### HR data reporting and dissemination

Most of the institutions did not have standardized HR data reporting forms available. Only ten (15%) institutions had some software to maintain HR data. The government has been supporting the development of modern HR software, because the one in use, does not generate any analytical report. In 62 (95%) institutions, a sample of data to be collected, was not available. Only 18 (28%) institutions used the software to disseminate the data, on as per required basis; others relied on the paper based system. In only nine institutions (14%), the respondents were satisfied with the performance of the software (Table 4).

**Table 4:** HR Data reporting and dissemination (n=65)

Standardized data reporting forms available	15%
Software capable of producing analytical reports	11%
Satisfaction with the performance of software	14%
Samples of analyzed data if available	5%
Support for developing HRIS software	
• Government	62%
• Donors	29%
• Own initiative	9%
HR data transmission mode	
• Paper	55%
• Specific software	28%
• Spread sheet	15%
• Online	2%
HR data transmission schedule	
Weekly	22%
Monthly	26%
Annually	3%
As required	49%

### Human resource data use

Only three out of 65 (5%) institutions used the HR data regularly for decision making, planning and management for human resources. In 50% of the institutions, HR data is not demanded at all by the managers, for any decision-making, whatsoever. Similarly, 60% institutions did not have categories or cadres shown in their data. In 85% of institutions, HR codes did not exist (Table 5).

**Table 5:** Use of human resource data by the institutions (n=65)

HR cadre list available	40%
HR codes in use	15%
Demand for HR data for decision making	55%
Main user of HR data	
• Facility in-charge	50%
• HR manager	20%
• Higher authorities	25%
HR data use in decision making, planning, management	
• Regularly	5%
• Intermittently	35%
• As required	60%

### HRIS monitoring

Twenty percent of the institutions had a professional staff responsible for maintaining the HRIS. In 41 (63%) institutions, it was the Director General Health who is responsible to monitor the HRIS. The monitoring again was not regular in most institutions, and it was done on as per required basis (73%). The feedback channel was found in only 20% of the cases (Table 6).

**Table 6:** HRIS monitoring (n=65)

Any authority or committee to monitor HRIS	20%
Type of authority monitoring HRIS	
• DG Health	63%
• DHIS manager	17%
• Head of HRIS cell	20%
Frequency of HRIS monitoring	
• Weekly	2%
• Monthly	8%
• Annually	17%
• As and when required	73%
Feedback on HRIS	20%
Feedback authority	
• Local administration	58%
• Higher ups	17%
• No feedback	25%

## Discussion

Use of evidence has seldom been practiced for crucial decision making in Pakistan’s health sector [13]. The overall HRIS assessment revealed a very bleak picture. The main weaknesses found were inadequate IT facilities and lack of trained manpower; weak supporting and monitoring mechanism, negligible budget and almost no maintenance. In the first instance, these issues seem to be a common phenomenon of the developing countries where majority of the health systems are undergoing transition. Very few studies could be found for comparison. Nevertheless, one global review of HRIS describes the findings similar to those documented by our study and concludes that HRIS practices are fragile and weak within countries because of shortage of trained human resource in health care sector [14]. Management of human resources in health sector is dependent on the timely evidence generated by an efficient, modern and functional HRIS [15]. One of the stewardship roles of the provincial governments is to build the capacity of their HRH and subsequently sustain them, to make the health system responsive to the needs of the population served [16]. The use of information technology in the management of health care workforce has been slow to start in most developing countries; however, the usefulness of the HRIS and its benefits in terms of better performance of any system has been realized with the advent of computers [17]. Many developing countries have benefited with the use of computer databases in the health sector and with the generation of evidence, which has eventually been utilized for effective strategic planning for an improved health system performance [18]. Continuing education and capacity-building initiatives for health providers are imperative for improving health care delivery in a highly pluralistic health care system in Pakistan [19]. This would be possible, only if a functional HRIS is in operation. Needless to say, that a well-developed strategic plan would provide a solid foundation for the development of a responsive health information system in Pakistan.

After the recent devolution, Pakistan does not have a national health ministry anymore, and all of the HR related functions have been devolved to the provinces. Therefore, there is a dire need to improve the reporting mechanism and data transmission from the grass root level to provide credible evidence to the provincial level for policy formulation and sectoral planning. Private organizations are still not sharing their data with public sector that creates an ambiguous state of affairs vis-à-vis actual HR number and skill mix.. It is well known that the private sector has well-trained staff with modern IT fcilities, and therefore it reflects relatively better planning, management and coordination. Public sector departments must learn from the examples and try to adapt the models.

## Conclusion

Development of a comprehensive and decentralized HRIS will carry the potential of a concerted monitoring and evaluation of the HR related indicators across the health sector in the provinces. In view of the gaps and flaws across the departments, recorded in our study, we conclude that a well-established HRIS will facilitate data processing, report generation, transmission and feedback. Moreover, the provincial governments will be better prepared to face the challenges in order to meet the targets of the Millennium Development Goals. Given Pakistan’s devolved health care system and responsibility for health service provision, it is essential that provincial governments have a functional HRIS capable of producing accurate workforce analyses, facilitating health sector planning, and enabling the development of evidenced-based HR policies.

## Competing interests

The authors declare that they have no competing interests.

## Authors’ contribution

RK, BTS and JA designed the study. The work of data collection and database construction was distributed equally amongst RK, JA and SZ. Data analysis and interpretation was done by RK, BTS and JA. Provisional drafts of manuscript were written by RK and BTS. Intellectual inputs were provided by ZK, SM and MIM. Final revisions of draft were done by RK and BTS. All authors read and approved the final manuscript.

## Pre-publication history

The pre-publication history for this paper can be accessed here:

http://www.biomedcentral.com/1472-6947/13/104/prepub

## References

[B1] World Health Organization World Health Report 2006 2006 Geneva: Working together for health

[B2] Qazi MS Ali M Health Management Information System utilization in Pakistan: challenges, pitfalls and the way forward Biosci Trends 2011 5 6 245 254 22281538 10.5582/bst.2011.v5.6.245

[B3] Qazi MS Ali M Kuroiwa C The health management information system of Pakistan under devolution: health managers' perceptions Biosci Trends 2008 2 2 75 80 20103905

[B4] Siddiqi S Masud TI Nishtar S Peters DH Sabri B Bile KM Framework for assessing governance of the health system in developing countries: gateway to good governance Health Policy 2009 90 1 13 25 10.1016/j.healthpol.2008.08.005 18838188

[B5] Hafeez A Khan Z Bile KM Jooma R Sheikh M Pakistan human resources for health assessment, 2009 East Mediterr Health J 2010 16 Suppl S145 151 21495600

[B6] Dayrit MM Dolea C Dreesch N Addressing the Human Resources for Health crisis in countries: How far have we gone? What can we expect to achieve by 2015? Rev Peru Med Exp Salud Publica 2011 28 2 327 336 10.1590/S1726-46342011000200022 21845315

[B7] Qazi MS Ali M Pakistan's health management information system: health managers' perspectives J Pak Med Assoc 2009 59 1 10 14 19213369

[B8] Nishtar S The Gateway Paper–proposed health reforms in Pakistan–interface considerations J Pak Med Assoc 2006 56 12 Suppl 4 S78 93 17595835

[B9] Eastern Mediterranian Regional Health System Observatory 2012 Pakistan: Health Systems Profiles [cited 18^th^ december 2012]; Available from: http://gis.emro.who.int/HealthSystemObservatory/Profile/Forms/frmProfileSelectionByCountry.aspx

[B10] World Health Organization 2007 Pakistan. Cairo: Report of the Health System Review Mission

[B11] Mazhar A Shaikh BT Reforms in Pakistan: Decisive times for improving maternal and child health Healthcare Policy 2012 8 1 24 32 23968601 PMC3430152

[B12] Management Sciences for Health Human resource management rapid assessment tool for public and private sector health organizations 2005 A guide for strengthening HRM System. Cambridge: A guide for strengthening HRM System

[B13] Yasir I Shaikh BT Use of evidence for decision making: A qualitative exploratory study on MNCH Program Pakistan: Pak J Public Health

[B14] Riley P Zuber A Vindigni S Gupta N Verani A Sunderland N Information systems on human resources for health: a global review Hum Resour Health 2012 10 7 10.1186/1478-4491-10-7 22546089 PMC3433380

[B15] Fagerstrom L Evidence-based human resource management: a study of nurse leaders' resource allocation J Nurs Manag 2009 17 4 415 25 10.1111/j.1365-2834.2009.01010.x 19531141

[B16] Shaikh BT Kadir MM Hatcher J Health care and Public health in South Asia Public Health 2006 120 2 142 144 10.1016/j.puhe.2005.08.018 16330057

[B17] Tansley C Watson T Strategic exchange in the development of Human Resource Information Systems (HRIS): New Technology Work and Employment 2000 15 2 108 122 10.1111/1468-005X.00068

[B18] Spero JC McQuide PA Matte R Tracking and monitoring the health workforce: a new human resources information system (HRIS) in Uganda Hum Resour Health 2011 9 6 10.1186/1478-4491-9-6 21329516 PMC3049180

[B19] Shaikh BT Rabbani F Rahim M Health workers for change: a tool for promoting behavior change among health providers East Mediterr Health J 2006 12 3/4 331 339 17037702

